# Examining Hope in Adolescents with Chronic Musculoskeletal Pain

**DOI:** 10.3390/children13040457

**Published:** 2026-03-27

**Authors:** Irene Chern, Nellie Butler, Mackenzie McGill, Rui Xiao, Peter F. Cronholm, Jami F. Young, Tonya M. Palermo, Pamela F. Weiss, Abby R. Rosenberg, Sabrina Gmuca

**Affiliations:** 1 Department of Pediatrics, Division of Rheumatology, Seattle Children’s Hospital, Seattle, WA 98105, USA; ichern@uw.edu; 2 Department of Pediatrics, Division of Rheumatology, Children’s Hospital of Philadelphia, Philadelphia, PA 19104, USA; butlern2@chop.edu (N.B.); mcgillm1@chop.edu (M.M.); weisspa@chop.edu (P.F.W.); 3 Clinical Futures, Children’s Hospital of Philadelphia, Philadelphia, PA 19146, USA; 4 PolicyLab, Children’s Hospital of Philadelphia, Philadelphia, PA 19146, USA; youngjf@chop.edu; 5 Department of Biostatistics, Epidemiology and Informatics, Perelman School of Medicine, University of Pennsylvania, Philadelphia, PA 19104, USA; xiaor@chop.edu; 6 Department of Family Medicine and Community Health, Perelman School of Medicine, University of Pennsylvania, Philadelphia, PA 19104, USA; peter.cronholm@pennmedicine.upenn.edu; 7 Leonard Davis Institute of Health Economics, University of Pennsylvania, Philadelphia, PA 19104, USA; 8 Department of Psychiatry, Perelman School of Medicine, University of Pennsylvania, Philadelphia, PA 19104, USA; 9 Department of Child and Adolescent Psychiatry and Behavioral Sciences, Children’s Hospital of Philadelphia, Philadelphia, PA 19104, USA; 10 Department of Anesthesiology and Pain Medicine, University of Washington School of Medicine, Seattle, WA 98195, USA; tonya.palermo@seattlechildrens.org; 11 Center for Child Health, Behavior and Development, Seattle Children’s Research Institute, Seattle, WA 98101, USA; 12 Department of Pediatrics, Perelman School of Medicine, University of Pennsylvania, Philadelphia, PA 19104, USA; 13 Department of Supportive Oncology, Division of Pediatric Palliative Care, Dana-Farber Cancer Institute, Boston, MA 02215, USA; abbyr_rosenberg@dfci.harvard.edu; 14 Department of Pediatrics, Boston Children’s Hospital, Boston, MA 02115, USA; 15 Department of Pediatrics, Harvard Medical School, Boston, MA 02115, USA

**Keywords:** psychosocial factors, psychological resilience, sense of agency, patient-reported outcomes

## Abstract

**Highlights:**

**What are the main findings?**

- Adolescents with chronic musculoskeletal pain reported being only slightly hopeful.
- Hope was associated with various measures of physical, mental, and overall health in this patient population.

**What are the implications of the main finding?**

- Interventions targeting hope and resilience may have the potential to bolster treatment outcomes and overall health in youth affected by chronic pain.
- Further research should explore the efficacy of cognitive behavioral therapy-based interventions, value clarification exercises, physical activity, support groups, and other methods to improve hope in youth with chronic pain.

**Abstract:**

Background/Objectives: This cross-sectional study aimed to quantify hope levels in adolescents with chronic musculoskeletal pain (CMP) and examine patient-reported outcomes associated with hope. Methods: This was an exploratory, cross-sectional, secondary analysis of baseline data from a prospective, single-center longitudinal study of 60 youth presenting for an initial evaluation at a pediatric subspecialty pain clinic. Subjects were English-speaking 12–17-year-olds with a diagnosis of CMP, primarily female and non-Hispanic White, with diffuse pain, median pain duration of 2 years, and moderate to severe physical dysfunction. Subjects completed surveys measuring hope (Children’s Hope Scale [CHS]) and patient-reported mental, physical, and overall health. Associations between hope scores (total and each subscale) and patient-reported outcomes were evaluated using Spearman rank correlations. Results: The median CHS score was 20.0 (IQR: 16.5–25.0), indicating slight hope. Patient hope was negatively correlated with depression (r = −0.61), anxiety (r = −0.49), psychological distress (r = −0.52), functional disability (r = −0.43), and pain interference (r = −0.37), but not pain intensity. Adolescents’ hope was positively correlated with resilience (r = 0.74) and overall health (r = 0.55; all *p* < 0.01). Conclusions: Hope is correlated with various patient-reported health measures in youth with CMP. Although causal inferences are not possible due to the cross-sectional nature of this study, the results suggest that hope may be an important coping mechanism in pediatric chronic pain. Future efforts to incorporate existing resilience coaching programs into usual care may improve hope and health-related quality of life in youth with CMP.

## 1. Introduction

According to a recent meta-analysis, chronic musculoskeletal pain (CMP) impacts more than one in four children and adolescents [1]. Chronic musculoskeletal pain negatively impacts children’s quality of life and physical and psychosocial functioning across wide-ranging domains. Youth with CMP have impaired school attendance, family relationships, mental health, and higher rates of suicidal thoughts and behaviors [2,3,4,5,6]. Considering the reduced quality of life of many young people with CMP, contributors to psychosocial wellbeing and coping skills in this population warrant attention.

Hope, defined by Snyder as ‘the perceived capability to derive pathways to desired goals and motivate oneself via agency thinking to use those pathways [7],’ is positively associated with pediatric health. Previous studies suggest that hope is positively correlated with academic performance [8], quality of life [9,10], active coping [11,12,13], and medication adherence [14,15]. Prior work also suggests that low hope is correlated with psychosocial distress, including depression, anxiety, and suicidality in youth [16,17]. Furthermore, children with chronic illness report lower total hope scores in comparison to peers without chronic illnesses [18].

Hope has been an instrumental concept in the positive-psychology movement for over three decades. A positive motivational state, hope is characterized by the perceived capability to set desired goals, derive pathways to achieve them, and motivate oneself through agency thinking to pursue those pathways [19,20]. Hope is an important coping mechanism, affecting how individuals respond to stressful or life-threatening situations, thereby contributing to resilience and preventing psychological distress [20]. Prior studies have shown hope to be a quantifiable entity [19,21,22]. Validated measures of hope have been used across a wide range of settings to understand the responses of children and adolescents experiencing adversity ranging from treatment for cancer to those affected by war [23,24].

Levels of hope have not been explored specifically in youth affected by chronic pain. In an effort to reduce the substantial disability associated with CMP, we aim to assess whether hope should be further explored as a possible target for intervention to improve the quality of life and treatment outcomes of adolescents with chronic pain. Because interdisciplinary treatment for CMP involves engaging in multiple concurrent therapies (physical therapy, occupational therapy, and/or psychological counseling) and involves intensive physical exercise [25,26,27,28], hope about the positive impacts of treatment may be an important factor motivating patients to participate in these challenging therapies. Furthermore, hope is an accessible term that most adolescents are already familiar with. As such, hope appears to be a suitable target for further exploration in this population.

The aims of this study were to (1) quantify hope levels in adolescents with CMP, and (2) examine demographics, clinical characteristics, and patient-reported outcomes associated with hope. We hypothesized that adolescents with CMP, like those with other chronic illnesses, would have low hope and that hope would be correlated with mental, physical, and overall health outcomes [29].

## 2. Materials and Methods

### 2.1. Study Population

This was an exploratory, cross-sectional, secondary analysis of baseline data from a prospective, single-center longitudinal study of 60 patients seen for an initial evaluation at a subspecialty pain clinic at a tertiary care pediatric hospital from September 2021 through May 2023. The research protocol was approved by the local Institutional Review Board (IRB 21-019156).

### 2.2. Inclusion Criteria

Eligible patients were between 12 and 17 years old and diagnosed with CMP, defined as bone, joint, muscle, or soft-tissue pain lasting greater than or equal to three months by the treating physician. Specifically, eligibility was limited to patients diagnosed with localized or diffuse amplified musculoskeletal pain syndrome (AMPS). AMPS was defined as pain that is excessive relative to the triggering stimulus and occurs in the absence of an identifiable medical cause, such as inflammation [30]. Localized AMPS describes patients with pain in <5 body areas, while diffuse AMPS describes those with pain in ≥5 body areas [31]. Patients were required to read or understand English well enough to complete study assessments, guardians had to provide written informed consent for the child subjects, and children needed to assent to participation.

### 2.3. Exclusion Criteria

Patients with localized head pain, localized abdominal pain, or complex regional pain syndrome (CRPS) type I were excluded. We excluded patients with CRPS with the intent of studying a cohort of children and adolescents with chronic idiopathic pain in the absence of autonomic changes. We did not exclude or systematically assess for those with comorbid inflammatory rheumatic disease, hypermobility, or other collagen/connective-tissue disorders. Patients whose current medical status or cognitive functioning precluded completion of the assessment instruments were also excluded.

### 2.4. Study Measures

At the time of the initial study visit (initial clinic evaluation), patients received a series of questionnaires which were designed to be completed within approximately 4–6 weeks of their initial clinic evaluation. Surveys included demographics; hope measures; and patient-reported mental, physical, and overall health measures. Survey data were complemented by retrospective review of patient charts for clinical characteristics including pain-related measures, self-reported psychological history, and family and caregiver health history.

### 2.5. Data Analysis

Patient demographics and clinical characteristics were summarized by median and interquartile range (IQR) for continuous variables, and frequency and percentage for categorical variables. Medians and IQRs were selected due to the non-normal distribution of most variables; however, hope scores were also described using means and standard deviations due to their (roughly) normal distributions. Spearman rank correlation coefficients were calculated to examine the correlation between patients’ CHS scores (total, pathways subscores, and agency subscores, respectively) and variables of interest. The correlation coefficients of 0.8 or higher were considered as very strong, 0.55 to 0.79 as moderately strong, 0.30 to 0.54 as fair, and less than 0.30 as poor [32]. Because this was a secondary analysis of baseline data from a longitudinal study, this analysis was not sufficiently powered to analyze associations related to hope, and no formal adjustment for multiple comparisons was applied. Findings should be considered exploratory. Exclusions of missing data were performed on a variable-by-variable basis. All analyses were completed using Stata (version 18.0; StataCorp; College Station, TX, USA). Two-sided *p*-values less than 0.05 were considered statistically significant.

### 2.6. Patient Survey Measures

The Children’s Hope Scale (CHS) [33] was developed by Snyder and colleagues as a trait hope measure for children assessing hopeful patterns of thinking. This scale consists of six questions where children self-assign a score between 1 (none of the time) and 6 (all of the time) for a total possible score range between 6 and 36. Three questions in the scale assess agency thinking, while three assess pathways thinking. Scores were categorized as low hope (6–12 points), slight hope (13–23 points), moderate hope (24–29 points), and high hope (30–36 points). This scale exhibits good internal consistency, with Cronbach’s α ranging from 0.72 to 0.86, with a median α of 0.77, across samples of typically developing children and those with physical and mental health conditions [33].

The Patient Health Questionnaire-8 (PHQ-8) [34] is a modified version of the Patient Health Questionnaire-9 (PHQ-9) that omits the final question on suicidality. The PHQ-9 was created as a patient self-administered screening for depression [34]. The PHQ-8 consists of eight questions regarding symptoms of depression scored from 0 (not at all) to 3 (nearly every day) for a total possible score range of 0 to 24 points. Scores on the PHQ-8 were classified as no or minimal depression (0–4 points), mild depression (5–9 points), moderate depression (10–14 points), moderately severe (15–19 points), and severe depression (20–24 points). The PHQ-8 demonstrates strong internal consistency, with Cronbach’s α of 0.87 in a large European sample [35].

The Generalized Anxiety Disorder 7 (GAD-7) [36] is a patient-administered screen for generalized anxiety, which has been validated in a wide range of settings. The GAD-7 consists of seven questions regarding symptoms of generalized anxiety rated from 0 (not at all) to 3 (nearly every day) for a total possible score range of 0–21 points. Scores on the GAD-7 were classified as minimal anxiety (0–4 points), mild anxiety (5–9 points), moderate anxiety (10–14 points), and severe anxiety (15–21 points). This scale has excellent internal consistency, with a Cronbach’s α of 0.92 [36].

The Kessler-6 Psychological Distress Scale (K6) [37,38] is a self-report questionnaire measuring general psychological distress. The K6 consists of six questions regarding feelings of psychological distress and their impact on day-to-day functioning. Questions are coded as a score of 0 (none of the time) to 4 (all of the time) for a possible composite score of 0–24. Internal consistency is high (Cronbach’s α = 0.89) [38].

The 10-item Connor–Davidson Resilience Scale (CD-RISC-10) [39] is an abbreviated version of the original 25-item Connor–Davidson Resilience Scale [40], which has been validated across many settings. The scale includes 10 questions assessing resilience. Possible responses for each question range from a score of 0 (not at all true) to 4 (true nearly all of the time) for a total possible score of 0 to 40. Scores in the general adult population have a median of 32 (IQR: 29–36). There are currently no accepted score cutoff points for the adolescent population. With a Cronbach’s α of 0.85, the CD-RISC-10 demonstrates good internal consistency [39].

The Functional Disability Inventory (FDI) [41] is a tool designed to assess the impact of physical disability on an individual’s daily activities. The FDI includes 15 questions that assess limitations in mobility, self-care, and participation in social activities. Responses for each item range from 0 (no difficulty) to 4 (unable to do), with total scores ranging from 0 to 60. Higher scores indicate greater functional disability. Scores can be classified as no/minimal disability (0–12 points), moderate disability (13–29 points), and severe disability (30–60 points) [42]. The FDI has shown high internal consistency (Cronbach’s α = 0.85–0.92) [41].

The Patient-Reported Outcomes Measurement Information System (PROMIS) Pediatric Numeric Rating Scale v1.0-Pain Intensity [43] is a single self-report item from the PROMIS Pediatric-25 Profile v1.0 measuring pain intensity for children ages 8–18 years old with chronic pain. Respondents respond to the following question, ‘In the past 7 days, how bad was your pain on average?’ using a score between 0 (no pain) and 10 (worst pain imaginable).

The PROMIS Pediatric Bank v2.0-Pain Interference Short Form 8a (PROMIS Pain Interference SF-8a) [44] is an abbreviated version of the original 41-item PROMIS Pain Interference item bank [45]. This short form includes 8 items designed to assess the degree to which pain has interfered with an individual’s daily activities, such as schoolwork, leisure, and movement, within the past 7 days. Possible responses for each item range from 1 (never) to 5 (almost always). Raw scores are converted to T-scores, with a T-score of 50 representing the mean and a standard deviation of 10 for a sample including healthy youth and those with chronic illnesses [44]. As per guidance from the National Institutes of Health (NIH)-developed HealthMeasures resource, T-scores < 50 were considered within normal limits; 50–55, mild; 56–65, moderate; and >65, severe [46]. Cronbach’s α for Pain Interference raw scores was between 0.88 and 0.97 in a previous study [47].

The PROMIS Pediatric Global Health 7 (PGH-7) [48] is a 7-item self-report assessment of the overall health of children and adolescents, including general, physical, mental, and social domains. Possible responses for each item range from 1 (‘never’ or ‘poor’) to 5 (‘always’ or ‘excellent’). Raw scores are converted to T-scores, with a T-score of 50 representing the mean for the general United States population with a standard deviation of 10 [32]. As per guidelines from HealthMeasures, T-scores ≥ 42 were considered good; 37–41, fair; and ≤36, poor [46]. In a previous study, Cronbach’s α was reported as 0.87, indicating good internal consistency [49].

## 3. Results

Sixty patients were included in the final analyses. The median time to complete surveys was 3.43 (IQR: 2.71–4.86) weeks from the initial clinic evaluation. Demographic and clinical characteristics of patients are shown in Table 1. Patients were primarily female and Non-Hispanic White, with moderate to severe physical disability and widespread pain with a substantial disease duration of 2 years. The sample ranged from 12 to 17 years of age, with a mean of 15 years. Most patients had a history of anxiety or depression, and over half had family history of an anxiety or mood disorder. However, most patients had no history of adverse childhood experiences.

On average, patients were slightly hopeful, with a mean CHS score of 20.7 (SD: 6.4). Over half of patients were only slightly hopeful, while about one-third had moderate hope. Patient-reported outcome measures are shown in Table 2. See Appendix A for a descriptive stratification of patient-reported outcomes by hope level.

Co-occurring self-reported mental health conditions were common among the cohort. The sample overall had moderate depression and mild anxiety. Over one-third of patients demonstrated significant psychological distress on the K6. The median resilience score was near the middle of the range of possible scores on the CD-RISC-10. The study cohort also had significantly worse physical and overall health compared to the general adolescent population. Functional disability and pain interference were moderate, and half of the patients had pain intensity scores of at least seven out of ten. Overall, patients also reported poor health on the PGH-7.

**Figure 1:**
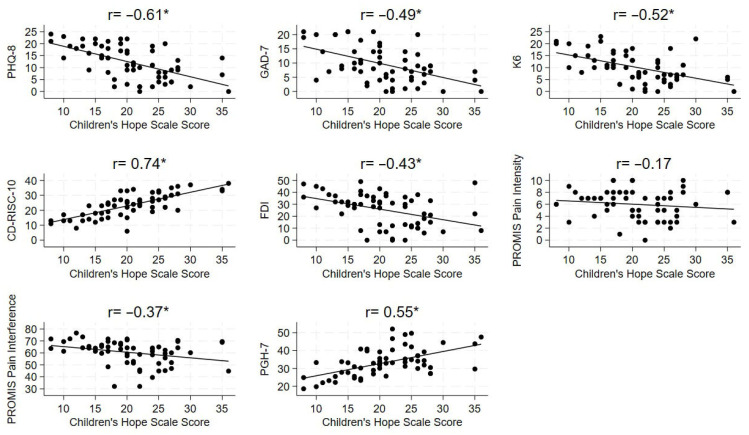
Correlation between Children’s Hope Scores and patient-reported health measures. Linear trend lines are descriptive only. Of note, the *y*-axis scales of each graph vary depending on the measure being analyzed. * Denotes *p* < 0.01 for Spearman rank correlation coefficient. Abbreviations: PHQ-8, Patient Health Questionnaire-8; GAD-7, Generalized Anxiety Disorder 7; K6, Kessler-6 Psychological Distress Scale; CD-RISC-10, 10-item Connor–Davidson Resilience Scale; FDI, Functional Disability Inventory; PROMIS, Patient-Reported Outcomes Measurement Information System; PGH-7, PROMIS Pediatric Global Health.

In the study cohort, CHS scores were significantly correlated with all patient-reported mental health measures (Figure 1). Total hope scores were negatively correlated with depression (r = −0.61), anxiety (r = −0.49), and psychological distress (r = −0.52), and positively with resilience (r = 0.74). The pathway and agency scores were also separately correlated with all mental health measures, with correlations ranging from fair to moderately strong (all *p* < 0.01). Hope pathway and agency scores had negative correlations with depression (r = −0.51 and r = −0.59, respectively) and anxiety (r = −0.37 and r = −0.51, respectively). Psychological distress was negatively correlated with pathways (r = −0.42) and agency (r = −0.52), while resilience was positively correlated with pathways (r = 0.72) and agency scores (r = 0.66).

In addition, Figure 1 shows correlations between patient hope levels and patient-reported physical and global health measures. Functional disability was negatively correlated with total hope scores (r = −0.43), as well as both subscales. Hope pathway scores had poor negative correlations with functional disability (r = −0.28; *p* < 0.05), while the correlation with agency scores was fair (r = −0.48; *p* < 0.01). Hope scores were not correlated with pain intensity (r = −0.16, r = −0.05, r = −0.23; for total hope, pathways, and agency, respectively). Although hope pathways scores were not correlated with pain interference, both total patient hope (r = −0.37) and agency scores (r = −0.40; all *p* < 0.01) were fairly negatively correlated with pain interference. Patients’ total hope (r = 0.55) and agency scores (r = 0.57) showed a moderately strong positive correlation with global health, while correlation with the pathways scores was fair (r = 0.46; all *p* < 0.01).

## 4. Discussion

This study demonstrated that adolescents with CMP completing an initial evaluation at a specialized pain clinic had lower than average adolescent population levels of hope, with exploratory findings suggesting an association between hope and patient-reported health outcomes in adolescents with CMP. The findings suggest that higher hope scores are associated with less depression, anxiety, psychological distress, and higher resilience. Greater hope may also be associated with lower functional disability and better overall health.

Patient hope levels in this study were lower than those found in previous studies of children and adolescents in the general population [33] and with medical conditions [51,52]. The mean total hope score in this study was lower than the reported average in a large sample of students in an urban Ohio district [53] and even notably lower than that of children and adolescents who had previously received cancer treatment [23]. Overall, this suggests that youth with CMP have hope scores comparable to, or lower than, children facing other significant chronic medical conditions.

The difference in correlation strength of the two hope subscores (pathways and agency) with patient-reported health outcomes warrants further investigation. Our exploratory findings suggest that hope agency scores have a stronger correlation with several health measures than hope pathways scores. This difference was particularly evident for functional disability and pain interference, despite the lack of correlation between hope scores and pain intensity. This suggests that among patients facing similar degrees of pain, those with higher agency may be better able to maintain physical and psychosocial functioning. Given that maintaining function is a major focus of treatment of CMP in children, interventions to increase agency may benefit this population. Increased agency has been associated with improved outcomes including reduced mental health comorbidity [11,12,13] and better treatment adherence for pediatric chronic diseases [9,15].

Perhaps unsurprisingly, the results suggest a moderately strong correlation between hope and resilience in this sample. This exploratory finding is logical given the similarity between these constructs, as evidenced by the content of the two scales. The themes of tenacity and control measured by the CD-RISC-10 correspond to the CHS agency subscale, while CD-RISC-10 items measuring decision-making under stress are reminiscent of the CHS pathways subscale [39]. Like hope, resilience levels were lower than average in this study, even compared to adolescents with other chronic illnesses [54,55]. This finding reinforces previous work showing low to moderate resilience in adolescents with CMP [56]. The possible associations seen between hope and patient-reported outcomes resemble prior research showing correlations between resilience and various health measures in adolescents with CMP, including physical disability and health-related quality of life [56].

A prior systematic review found that higher hope was correlated with positive outcomes in physical health, mental health, and interpersonal domains that are also associated with resilience [57]. Some prior studies suggest that resilience has a stronger correlation with mental health outcomes in adults than does hope [58]. However, other studies examining adults who experienced adverse childhood events suggest that hope has a stronger correlation with improved mental health outcomes than resilience [59]. The explanatory model that is perhaps most useful in describing the relationship between hope and resilience is exemplified by a previous finding that high-trait-hope individuals demonstrated more effective emotional recovery and diminished stress reactivity [60], suggesting that hope (including pathways and agency thinking) may be an important component of resilience. Ultimately, resilience and hope (and its components) are modifiable traits and thus are fertile ground for further investigation to potentially improve health and quality of life outcomes for adolescents with CMP.

The slight hope levels of patients in this study align with previous research on thought patterns and beliefs in youth with CMP. Past work indicates that while their ability to form goals is typical, adolescents with CMP perceive greater barriers to achieving their goals than do youth without pain. In addition, rumination on negative thoughts is common among youth with CMP and is often triggered by pain [61]. Together, these factors may serve as barriers to hopeful thinking in this population. The current study points to the need for future research to determine if adolescents with CMP could benefit from targeted interventions to promote positive thought patterns and improve their sense of hope in achieving desired goals.

While our findings do not support a causal relationship, they are hypothesis-generating for future studies that could examine the possible impact of psychosocial interventions on hope in chronic pain. One such intervention is Promoting Resilience in Stress Management (PRISM), a brief, one-on-one resilience coaching program covering skills related to stress management, goal setting, reframing negative thoughts, and finding the positives. This intervention has been shown to positively impact resilience, mental health, and quality of life for adolescents with serious illnesses [62,63,64], with exploratory analyses pointing to similar improvements specifically for adolescents with CMP [65]. Similarly, other cognitive behavioral therapy-based interventions for pediatric chronic pain (including WebMAP [66] and the Comfort Ability Pain Management Workshop [67], among others) may provide relevant skills to bolster hope and to potentially improve pain and pain-related outcomes. In addition, previous studies suggest that a variety of interventions may strengthen adolescent agency and closely related traits. These include value clarification exercises [68], learning critical and creative thinking skills [69], engaging in challenging physical activity [70], and participating in support groups [71].

This study has limitations. No causal or temporal conclusions can be drawn from this exploratory cross-sectional study, such as changes in hope over the treatment course or the impact of hope on treatment outcomes. Participants generally completed outcome measures within 2 to 5 weeks of their intake clinic visit; however, hope scores and other measures may differ for patients at different stages of treatment. Although CMP generally affects adolescents, the results may not be generalizable to children with CMP who are younger than 12 years old, as the sample ranged from 12 to 17 years, or to younger adolescents (12–13 years), who were underrepresented in the sample. It is possible that the analyses were insufficiently powered to detect all significant relationships between variable pairs due to the modest sample size of 60 patients. Several factors limit the generalizability of the results. Because patients were recruited from a specialty amplified musculoskeletal pain syndrome clinic at a tertiary pediatric hospital, it is possible that the sample differed from the broader population of adolescents with CMP. Due to this referral bias, findings may not generalize to community or primary-care CMP populations. Further research is needed to validate these results across broader adolescent CMP populations. We also acknowledge that psychological distress, anxiety, and depression, as measured by the surveys in this study, are highly linked to one another. In fact, in secondary analyses, we found collinearity between psychological distress, anxiety and depression. We chose to include all three separate measures given that the K6 uses a 30-day timeframe, rather than a 2-week window, and asks about frequency of distress experiences, making it particularly sensitive to recent psychological strain.

The results point to an association between hope and several mental, physical, and overall health domains in adolescents with CMP. These findings add to the body of evidence suggesting that hope may be a component of resilience. Future research is needed to explore the potential applicability of existing resilience coaching programs targeting hope for patients with CMP and to inform implementation of these interventions to bolster health outcomes in this patient population.

## Figures and Tables

**Table 1:** Demographic and clinical characteristics of patients.

	Total Cohort (*n* = 60) ^1^
**Demographics**	
Sex: Female (*n* = 58) ^2^	51 (87.9)
Age, years	15 (14–16)
Non-Hispanic White (*n* = 58) ^3^	43 (74.1)
**Pain-Related Measures**	
Reported Duration of Pain, months (*n* = 56) ^4^	24 (11–36)
Average Daily Pain (0–10) ^5^	6.5 (4.0–8.0)
Functional Disability Index (patient report; 0–60)	29.5 (12.5–36)
Widespread Pain Index (0–19)	9 (4–15)
Symptom Severity Score (0–12)	7 (5–9)
Met Fibromyalgia Criteria (*n* = 58) ^6^	36 (62.1)
**Self-Reported Psychological History**	
Anxiety	49 (81.7)
Depression	32 (53.3)
Suicidality ^7^	11 (18.3)
Adverse Childhood Experience ^8^ Score: 0	41 (68.3)
Adverse Childhood Experience Score: 1 to 3	18 (30.0)
Adverse Childhood Experience Score: 4+	1 (1.7)
Family History of Anxiety or Mood Disorder ^9^	34 (56.7)

^1^ n (%); Median (IQR = interquartile range). ^2^ Sex was missing for 2 patients. ^3^ Race and ethnicity were missing for 2 patients. ^4^ Reported duration of pain was missing for 4 patients. ^5^ Average daily pain over the last 7 days. ^6^ Based on 2010 American College of Rheumatology criteria [50]. Missing for 2 patients. ^7^ History of suicidal ideation with intent or a suicide attempt. ^8^ Adverse childhood experience defined as: verbal abuse from parent/adult in household; physical abuse from parent/adult in household; sexual abuse by an adult or person at least 5 years older than patient; parent with an alcohol problem; parent with a drug problem; parents are divorced; parents are separated; other household members with problems with drugs or alcohol; household member has attempted/died by suicide; household member went to prison; family has economic hardship (e.g., not enough money for food, clothing, etc.); mother or step mother is a victim of domestic violence; or other. ^9^ History of anxiety, depression, or other mood disorder in at least one first-degree relative.

**Table 2:** Patients’ self-reported health measures.

	Total Cohort (*n* = 60)
**Hope Measures**	
CHS: total, median (IQR)	20.0 (16.5–25.0)
CHS: total, mean (SD)	20.7 (6.4)
CHS: agency, median (IQR)	9.5 (7.5–12.0)
CHS: agency, mean (SD)	9.8 (3.5)
CHS: pathways, median (IQR)	10.0 (8.5–13.0)
CHS: pathways, mean (SD)	10.8 (3.4)
**Mental Health Measures**	
PHQ-8, median (IQR)	11.5 (6.5–18.5)
GAD-7, median (IQR)	8.5 (4.5–14.0)
K6, median (IQR)	10.0 (6.0–14.0)
CD-RISC-10, median (IQR)	24.0 (17.5–29.5)
**Physical Health Measures**	
FDI, median (IQR)	29.5 (12.5–36.0)
PROMIS Pain Intensity, median (IQR)	6.5 (4.0–8.0)
PROMIS Pain Interference SF-8a, median (IQR)	63.4 (54.4–67.5)
**Overall Health Measures**	
PROMIS PGH-7, median (IQR)	32.9 (27.2–38.3)

Abbreviations: CHS, Children’s Hope Scale; PHQ-8, Patient Health Questionnaire-8; GAD-7, Generalized Anxiety Disorder 7; K6, Kessler-6 Psychological Distress Scale; CD-RISC-10, 10-item Connor–Davidson Resilience Scale; FDI, Functional Disability Inventory; PROMIS, Patient-Reported Outcomes Measurement Information System; SF-8a, Short Form 8a; PGH-7, PROMIS Pediatric Global Health.

## Data Availability

The raw data supporting the conclusions of this article will be made available by the authors on request. The data are not publicly available due to privacy/confidentiality restrictions of the participants.

## Appendix A

**Table A1:** Patients’ self-reported health measures, stratified by hope level ^1^.

	Total Cohort (*n* = 60) ^2^	Low Hope (*n* = 6 [10.0%]) ^2^	Slight Hope (*n* = 32 [53.3%]) ^2^	Moderate Hope (*n* = 18 [30.0%]) ^2^	High Hope (*n* = 4 [6.7%]) ^2^
**Mental Health Measures**					
PHQ-8	11.5 (6.5–18.5)	20.0 (18.0–23.0)	14.0 (9.0–19.0)	8.5 (4.0–11.0)	4.5 (1.0–10.5)
GAD-7	8.5 (4.5–14.0)	16.5 (7.0–20.0)	10.0 (5.5–15.0)	8.0 (5.0–11.0)	2.0 (0.0–5.5)
K6	10.0 (6.0–14.0)	17.5 (10.0–20.0)	11.0 (6.5–15.0)	7.0 (5.0–11.0)	5.5 (2.5–14.0)
CD-RISC-10	24.0 (17.5–29.5)	13.0 (11.0–13.0)	23.0 (17.0–25.5)	28.5 (22.0–32.0)	35.5 (33.5–37.5)
**Physical Health Measures**					
FDI	29.5 (12.5–36.0)	40.5 (36.0–45.0)	30.5 (17.5–36.5)	16.5 (11.0–30.0)	15.0 (7.5–35.0)
PROMIS Pain Intensity	6.5 (4.0–8.0)	6.5 (6.0–8.0)	7.0 (4.5–8.0)	5.0 (3.0–7.0)	7.0 (4.5–8.0)
PROMIS Pain Interference SF-8a	63.4 (54.4–67.5)	70.6 (63.7–71.8)	64.0 (55.2–66.8)	59.8 (51.2–64.1)	64.6 (52.5–69.2)
**Overall Health Measures**					
PROMIS PGH-7	32.9 (27.2–38.3)	22.6 (19.8–24.9)	32.3 (26.3–35.6)	34.7 (31.2–39.3)	44.15 (36.8–46.1)

^1^ Hope level subgroups are descriptive only. ^2^ Median (IQR). Abbreviations: PHQ-8, Patient Health Questionnaire-8; GAD-7, Generalized Anxiety Disorder 7; K6, Kessler-6 Psychological Distress Scale; CD-RISC-10, 10-item Connor–Davidson Resilience Scale; FDI, Functional Disability Inventory; PROMIS, Patient-Reported Outcomes Measurement Information System; SF-8a, Short Form 8a; PGH-7, PROMIS Pediatric Global Health.

## References

[1] Chambers C.T., Dol J., Tutelman P.R., Langley C.L., Parker J.A., Cormier B.T., Macfarlane G.J., Jones G.T., Chapman D., Proudfoot N. (2024). The prevalence of chronic pain in children and adolescents: A systematic review update and meta-analysis. Pain.

[2] Huguet A., Miró J. (2008). The severity of chronic pediatric pain: An epidemiological study. J. Pain.

[3] Roth-Isigkeit A., Thyen U., Stöven H., Schwarzenberger J., Schmucker P. (2005). Pain among children and adolescents: Restrictions in daily living and triggering factors. Pediatrics.

[4] Kashikar-Zuck S., Cunningham N., Sil S., Bromberg M.H., Lynch-Jordan A.M., Strotman D., Peugh J., Noll J., Ting T.V., Powers S.W. (2014). Long-Term Outcomes of Adolescents With Juvenile-Onset Fibromyalgia in Early Adulthood. Pediatrics.

[5] Kashikar-Zuck S., Ting T.V. (2014). Juvenile fibromyalgia: Current status of research and future developments. Nat. Rev. Rheumatol..

[6] van Tilburg M.A., Spence N.J., Whitehead W.E., Bangdiwala S., Goldston D.B. (2011). Chronic pain in adolescents is associated with suicidal thoughts and behaviors. J. Pain.

[7] Snyder C.R. (2002). Hope Theory: Rainbows in the Mind. Psychol. Inq..

[8] Day L., Hanson K., Maltby J., Proctor C., Wood A. (2010). Hope uniquely predicts objective academic achievement above intelligence, personality, and previous academic achievement. J. Res. Personal..

[9] Lloyd S.M., Cantell M., Pacaud D., Crawford S., Dewey D. (2009). Brief Report: Hope, Perceived Maternal Empathy, Medical Regimen Adherence, and Glycemic Control in Adolescents with Type 1 Diabetes. J. Pediatr. Psychol..

[10] Van Allen J., Seegan P.L., Haslam A., Steele R.G. (2016). Hope mediates the relationship between depression and quality of life among youths enrolled in a family-based pediatric obesity intervention. Child. Health Care.

[11] Santos S., Crespo C., Canavarro M.C., Kazak A.E. (2015). Family Rituals and Quality of Life in Children With Cancer and Their Parents: The Role of Family Cohesion and Hope. J. Pediatr. Psychol..

[12] Lewis H.A., Kliewer W. (1996). Hope, Coping, and Adjustment Among Children with Sickle Cell Disease: Tests of Mediator and Moderator Models. J. Pediatr. Psychol..

[13] Kliewer W., Lewis H. (1995). Family Influences on Coping Processes in Children and Adolescents with Sickle Cell Disease. J. Pediatr. Psychol..

[14] Venning A.J., Eliott J., Whitford H., Honnor J. (2007). The Impact of a Child’s Chronic Illness on Hopeful Thinking in Children and Parents. J. Soc. Clin. Psychol..

[15] Berg C.J., Rapoff M.A., Snyder C.R., Belmont J.M. (2007). The relationship of children’s hope to pediatric asthma treatment adherence. J. Posit. Psychol..

[16] Dori G.A., Overholser J.C. (1999). Depression, hopelessness, and self-esteem: Accounting for suicidality in adolescent psychiatric inpatients. Suicide Life Threat. Behav..

[17] Kazdin A.E., French N.H., Unis A.S., Esveldt-Dawson K., Sherick R.B. (1983). Hopelessness, depression, and suicidal intent among psychiatrically disturbed inpatient children. J. Consult. Clin. Psychol..

[18] Martins A.R., Crespo C., Salvador Á., Santos S., Carona C., Canavarro M.C. (2018). Does Hope Matter? Associations Among Self-Reported Hope, Anxiety, and Health-Related Quality of Life in Children and Adolescents with Cancer. J. Clin. Psychol. Med. Settings.

[19] Snyder C.R., Harris C., Anderson J.R., Holleran S.A., Irving L.M., Sigmon S.T., Yoshinobu L., Gibb J., Langelle C., Harney P. (1991). The will and the ways: Development and validation of an individual-differences measure of hope. J. Personal. Soc. Psychol..

[20] Alizadeh-Khanghahi M., Farshbaf-Khalili A., Alizadeh M., Jabraeili M. (2025). How supportive care needs influence resilience and hope in mothers of children with cancer?. BMC Pediatr..

[21] Snyder C.R., Sympson S.C., Ybasco F.C., Borders T.F., Babyak M.A., Higgins R.L. (1996). Development and validation of the State Hope Scale. J. Personal. Soc. Psychol..

[22] Snyder C.R., Cheavens J.S., Michael S.T. (2005). Hope Theory: History and Elaborated Model. Interdisciplinary Perspectives on Hope.

[23] Germann J.N., Leonard D., Heath C.L., Stewart S.M., Leavey P.J. (2018). Hope as a Predictor of Anxiety and Depressive Symptoms Following Pediatric Cancer Diagnosis. J. Pediatr. Psychol..

[24] Haroz E.E., Jordans M., de Jong J., Gross A., Bass J., Tol W. (2017). Measuring Hope Among Children Affected by Armed Conflict: Cross-Cultural Construct Validity of the Children’s Hope Scale. Assessment.

[25] Sherry D.D., Brake L., Tress J.L., Sherker J., Fash K., Ferry K., Weiss P.F. (2015). The Treatment of Juvenile Fibromyalgia with an Intensive Physical and Psychosocial Program. J. Pediatr..

[26] Tran S.T., Thomas S., DiCesare C., Pfeiffer M., Sil S., Ting T.V., Williams S.E., Myer G.D., Kashikar-Zuck S. (2016). A pilot study of biomechanical assessment before and after an integrative training program for adolescents with juvenile fibromyalgia. Pediatr. Rheumatol. Online J..

[27] Tran S.T., Guite J.W., Pantaleao A., Pfeiffer M., Myer G.D., Sil S., Thomas S.M., Ting T.V., Williams S.E., Edelheit B. (2017). Preliminary Outcomes of a Cross-Site Cognitive-Behavioral and Neuromuscular Integrative Training Intervention for Juvenile Fibromyalgia. Arthritis Care Res..

[28] Odell S., Logan D.E. (2013). Pediatric pain management: The multidisciplinary approach. J. Pain Res..

[29] Chern I., McGill M., Butler N., Gmuca S. (2024). Hope and Health in Adolescents with Chronic Musculoskeletal Pain. Arthritis Rheumatol.

[30] Sherry D.D. (2008). Amplified musculoskeletal pain: Treatment approach and outcomes. J. Pediatr. Gastroenterol. Nutr..

[31] Sherry D.D., Sonagra M., Gmuca S. (2020). The spectrum of pediatric amplified musculoskeletal pain syndrome. Pediatr. Rheumatol. Online J..

[32] Chan Y.H. (2003). Biostatistics 104: Correlational analysis. Singap. Med. J..

[33] Snyder C.R., Hoza B., Pelham W.E., Rapoff M., Ware L., Danovsky M., Highberger L., Rubinstein H., Stahl K.J. (1997). The development and validation of the Children’s Hope Scale. J. Pediatr. Psychol..

[34] Wu Y., Levis B., Riehm K.E., Saadat N., Levis A.W., Azar M., Rice D.B., Boruff J., Cuijpers P., Gilbody S. (2020). Equivalency of the diagnostic accuracy of the PHQ-8 and PHQ-9: A systematic review and individual participant data meta-analysis. Psychol. Med..

[35] Arias de la Torre J., Vilagut G., Ronaldson A., Valderas J.M., Bakolis I., Dregan A., Molina A.J., Navarro-Mateu F., Pérez K., Bartoll-Roca X. (2023). Reliability and cross-country equivalence of the 8-item version of the Patient Health Questionnaire (PHQ-8) for the assessment of depression: Results from 27 countries in Europe. Lancet Reg. Health Eur..

[36] Spitzer R.L., Kroenke K., Williams J.B.W., Löwe B. (2006). A Brief Measure for Assessing Generalized Anxiety Disorder: The GAD-7. Arch. Intern. Med..

[37] Kessler R.C., Andrews G., Colpe L.J., Hiripi E., Mroczek D.K., Normand S.L.T., Walters E.E., Zaslavsky A.M. (2002). Short screening scales to monitor population prevalences and trends in non-specific psychological distress. Psychol. Med..

[38] Kessler R.C., Barker P.R., Colpe L.J., Epstein J.F., Gfroerer J.C., Hiripi E., Howes M.J., Normand S.-L.T., Manderscheid R.W., Walters E.E. (2003). Screening for Serious Mental Illness in the General Population. Arch. Gen. Psychiatry.

[39] Campbell-Sills L., Stein M.B. (2007). Psychometric analysis and refinement of the Connor-davidson Resilience Scale (CD-RISC): Validation of a 10-item measure of resilience. J. Trauma. Stress.

[40] Connor K.M., Davidson J.R.T. (2003). Development of a new resilience scale: The Connor-Davidson Resilience Scale (CD-RISC). Depress. Anxiety.

[41] Walker L.S., Greene J.W. (1991). The functional disability inventory: Measuring a neglected dimension of child health status. J. Pediatr. Psychol..

[42] Kashikar-Zuck S., Flowers S.R., Claar R.L., Guite J.W., Logan D.E., Lynch-Jordan A.M., Palermo T.M., Wilson A.C. (2011). Clinical utility and validity of the Functional Disability Inventory among a multicenter sample of youth with chronic pain. Pain.

[43] HealthMeasures PROMIS Numeric Rating Scale v1.0—Pediatric Pain Intensity 1a. https://www.healthmeasures.net/index.php?option=com_instruments&view=measure&id=898&Itemid=992.

[44] Varni J.W., Stucky B.D., Thissen D., Dewitt E.M., Irwin D.E., Lai J.S., Yeatts K., Dewalt D.A. (2010). PROMIS Pediatric Pain Interference Scale: An item response theory analysis of the pediatric pain item bank. J. Pain.

[45] Amtmann D., Cook K.F., Jensen M.P., Chen W.H., Choi S., Revicki D., Cella D., Rothrock N., Keefe F., Callahan L. (2010). Development of a PROMIS item bank to measure pain interference. Pain.

[46] HealthMeasures (2025). Score Cut Points for PROMIS^®^ Pediatric and Parent Proxy Measures. https://www.healthmeasures.net/score-and-interpret/interpret-scores/promis/promis-score-cut-points/promis-pediatric-and-parent-proxy-score-cut-points.

[47] Chen C.X., Kroenke K., Stump T.E., Kean J., Carpenter J.S., Krebs E.E., Bair M.J., Damush T.M., Monahan P.O. (2018). Estimating minimally important differences for the PROMIS pain interference scales: Results from 3 randomized clinical trials. Pain.

[48] Forrest C.B., Bevans K.B., Pratiwadi R., Moon J., Teneralli R.E., Minton J.M., Tucker C.A. (2014). Development of the PROMIS ^®^ pediatric global health (PGH-7) measure. Qual. Life Res..

[49] Luijten M.A.J., Haverman L., van Litsenburg R.R.L., Roorda L.D., Grootenhuis M.A., Terwee C.B. (2022). Advances in measuring pediatric overall health: The PROMIS^®^ Pediatric Global Health scale (PGH-7). Eur. J. Pediatr..

[50] Wolfe F., Clauw D.J., Fitzcharles M.A., Goldenberg D.L., Katz R.S., Mease P., Russell A.S., Russell I.J., Winfield J.B., Yunus M.B. (2010). The American College of Rheumatology preliminary diagnostic criteria for fibromyalgia and measurement of symptom severity. Arthritis Care Res..

[51] Calkins-Smith A.K., Marker A.M., Clements M.A., Patton S.R. (2018). Hope and mealtime insulin boluses are associated with depressive symptoms and glycemic control in youth with type 1 diabetes mellitus. Pediatr. Diabetes.

[52] Connelly T.W. (2005). Family functioning and hope in children with juvenile rheumatoid arthritis. MCN Am. J. Matern. Child. Nurs..

[53] Bean G.J. (2020). An Item Response Theory Analysis of the Children’s Hope Scale. J. Soc. Soc. Work Res..

[54] Rosenberg A.R., Yi-Frazier J.P., Eaton L., Wharton C., Cochrane K., Pihoker C., Baker K.S., McCauley E. (2015). Promoting Resilience in Stress Management: A Pilot Study of a Novel Resilience-Promoting Intervention for Adolescents and Young Adults With Serious Illness. J. Pediatr. Psychol..

[55] Carlsen K., Haddad N., Gordon J., Phan B.L., Pittman N., Benkov K., Dubinsky M.C., Keefer L. (2017). Self-efficacy and Resilience Are Useful Predictors of Transition Readiness Scores in Adolescents with Inflammatory Bowel Diseases. Inflamm. Bowel Dis..

[56] Gmuca S., Xiao R., Urquhart A., Weiss P.F., Gillham J.E., Ginsburg K.R., Sherry D.D., Gerber J.S. (2019). The Role of Patient and Parental Resilience in Adolescents with Chronic Musculoskeletal Pain. J. Pediatr..

[57] Ong A.D., Standiford T., Deshpande S. (2018). Hope and stress resilience. The Oxford Handbook of Hope; Oxford library of psychology.

[58] Morote R., Hjemdal O., Krysinska K., Martinez Uribe P., Corveleyn J. (2017). Resilience or hope? Incremental and convergent validity of the resilience scale for adults (RSA) and the Herth hope scale (HHS) in the prediction of anxiety and depression. BMC Psychol..

[59] Munoz R.T., Hanks H., Hellman C.M. (2020). Hope and resilience as distinct contributors to psychological flourishing among childhood trauma survivors. Traumatology.

[60] Ong A.D., Edwards L.M., Bergeman C.S. (2006). Hope as a source of resilience in later adulthood. Personal. Individ. Differ..

[61] Edwards M.J., Tang N.K., Wright A.M., Salkovskis P.M., Timberlake C.M. (2011). Thinking about thinking about pain: A qualitative investigation of rumination in chronic pain. Pain Manag..

[62] Rosenberg A.R., Bradford M.C., Barton K.S., Etsekson N., McCauley E., Curtis J.R., Wolfe J., Baker K.S., Yi-Frazier J.P. (2019). Hope and benefit finding: Results from the PRISM randomized controlled trial. Pediatr. Blood Cancer.

[63] Rosenberg A.R., Bradford M.C., McCauley E., Curtis J.R., Wolfe J., Baker K.S., Yi-Frazier J.P. (2018). Promoting resilience in adolescents and young adults with cancer: Results from the PRISM randomized controlled trial. Cancer.

[64] Rosenberg A.R., Fladeboe K.M., Zhou C., Bradford M.C., Kang T., Maurer S., Freyer D.R., Baker K.S., Comiskey L., Junkins C.C. (2025). Promoting Resilience in Stress Management: A Randomized Controlled Trial of a Novel Psychosocial Intervention for Adolescents and Young Adults With Advanced Cancer. JCO Oncol. Pr..

[65] Gmuca S., Weiss P.F., McGill M., Xiao R., Ward M., Nelson M., Sherry D.D., Cronholm P.F., Gerber J.S., Palermo T.M. (2022). The Feasibility and Acceptability of Resilience Coaching for Adolescent Chronic Musculoskeletal Pain: A Single-Arm Pilot Trial. Children.

[66] Palermo T.M., de la Vega R., Murray C., Law E., Zhou C. (2020). A digital health psychological intervention (WebMAP Mobile) for children and adolescents with chronic pain: Results of a hybrid effectiveness-implementation stepped-wedge cluster randomized trial. Pain.

[67] Coakley R., Wihak T., Kossowsky J., Iversen C., Donado C. (2017). The Comfort Ability Pain Management Workshop: A Preliminary, Nonrandomized Investigation of a Brief, Cognitive, Biobehavioral, and Parent Training Intervention for Pediatric Chronic Pain. J. Pediatr. Psychol..

[68] James M.R. (1990). Adolescent values clarification: A positive influence on perceived locus of control. J. Alcohol. Drug Educ..

[69] Flor R.K., Bita A., Monir K.C., Zohreh Z.Z. (2013). The Effect of Teaching Critical and Creative Thinking Skills on the Locus of Control and Psychological Well-being in Adolescents. Procedia-Soc. Behav. Sci..

[70] Holloway J.B., Beuter A., Duda J.L. (1988). Self-efficacy and training for strength in adolescent girls. J. Appl. Soc. Psychol..

[71] Vijayaraghavan J., Vidyarthi A., Livesey A., Gittings L., Levy M., Timilsina A., Mullings D., Armstrong C. (2022). Strengthening adolescent agency for optimal health outcomes. BMJ.

